# Clarifying the *Dioscorea buchananii* Benth. species complex: a new potentially extinct subspecies for South Africa

**DOI:** 10.3897/phytokeys.48.6806

**Published:** 2015-04-15

**Authors:** Paul Wilkin, A. Muthama Muasya

**Affiliations:** 1 Royal Botanic Gardens, Kew, Richmond, Surrey, TW9 3AB, UK; 2 Department of Botany, University of Cape Town, Private Bag X3, Rondebosch 7701, SOUTH AFRICA

**Keywords:** Africa, South Africa, *Dioscorea*, yam, species, taxonomy, vines, conservation, steroids, eMonocot scratchpad

## Abstract

The *Dioscorea buchananii* complex is shown to comprise three species, one of which is divided into two subspecies, based on morphological data. Two species, *Dioscorea rupicola* Kunth and *Dioscorea multiloba* Kunth, are endemic or subendemic to South Africa and of widespread occurrence in KwaZulu Natal. They differ markedly from each other in inflorescence and floral morphology and appear to be ecologically differentiated. The third species, *Dioscorea buchananii* Benth., is primarily found in southeastern tropical Africa, but a small number of specimens collected in South Africa in the late 19^th^ and early 20^th^ centuries are placed in an endemic subspecies, Dioscorea buchananii subsp. undatiloba (Baker) Wilkin. The latter taxon is a high priority in terms of rediscovery and conservation. Keys, descriptions, supporting information and illustrations are provided and made available online through eMonocot biodiversity informatics tools. Three nomenclatural acts are undertaken: two names are placed in synonymy and a new combination made.

## Introduction

The *Dioscorea buchananii* Benth. group of species first came to the attention of science through the publication of three species with palmately lobed leaves from South Africa that had been collected by some of the early botanical explorers of the Cape: *Dioscorea diversifolia* Kunth, *Dioscorea multiloba* Kunth and *Dioscorea rupicola* Kunth (Kunth 1850). The first of those names was unfortunately a homonym of *Dioscorea diversifolia* Griseb., a Brazilian species for which a *Sellow* collection was cited (Grisebach 1842), although Kunth linked his name to *Drège* 4497 from the Cape. Two specimens of *Dioscorea multiloba* were cited by Kunth, *Drège* 4495 and 4496; both it and *Dioscorea diversifolia* were grouped as Cape taxa within one of Kunth’s species groups based on floral morphological characters. *Dioscorea rupicola*, which was based on a plant cultivated in Berlin from material that had been received from Ecklon in South Africa, was treated separately, perhaps because it had 3, not 6 stamens unlike all its relatives. Baker (1897) recognised all 3 species described by Kunth and added a fourth, *Dioscorea undatiloba* Baker that he had described a few years earlier (Baker 1889) with pedicellate flowers and “repand-pinnatifid” central leaf lobes. They were separated in the key in the Flora Capensis treatment through pedicel presence or absence, stamen number and leaf shape. A further taxon, *Dioscorea junodii* Burtt-Davy from Shilouvane in what is now Limpopo province was described by Burtt-Davy (1924). It was said to differ from *Dioscorea rupicola* in possessing racemose male inflorescences with 6 stamens per flower and in its leaf shape. *Dioscorea junodii* was placed in synonymy with *Dioscorea sylvatica* Ecklon by Govaerts and Wilkin (2015). In tropical Africa, *Dioscorea buchananii* Benth. was described by Bentham (1882) based on a specimen from what is now Malawi with entire leaves and large flowers in a dense, short inflorescence. Bentham did not suggest any affinities for the species.

Knuth (1924) was the first taxonomist to formally group these species into an infrageneric taxon, Dioscorea sect. Rhacodophyllum Uline ex R.Knuth, following their placement as “*Eudioscoreae capenses*” by Uline (1897). He also described a further species, *Dioscorea digitaria* R.Knuth (Knuth 1924), based on a *Rudatis* specimen from Friedenau in what is now lowland coastal KwaZulu Natal. The Pflanzenreich account (Knuth 1924) stated that it differed in its narrow, acute central leaf lobe and shorter male inflorescence. Knuth’s key to Dioscorea sect. Rhacodophyllum is based on similar characters to those used by Baker. A further new species, *Dioscorea natalensis* R.Knuth was placed in separate section containing South African species with entire leaves (Knuth 1924), but its inflorescence and floral morphology unequivocally link it to the *Dioscorea rupicola* group. The Pflanzenreich treatment also noted that a newly described variety of *Dioscorea buchananii* with palmately lobed leaves, var. ukamensis Uline ex R. Knuth linked *Dioscorea buchananii* to *Dioscorea undatiloba* and that perhaps those two species would be better combined. Knuth was the last taxonomist to study both the tropical and South African taxa of the *Dioscorea rupicola* species group.

Archibald (1968) covered only Eastern Cape specimens and populations of *Dioscorea rupicola* in her account of Cape *Dioscorea*. Von Teichman (1975) surveyed all the species of *Dioscorea* in South Africa. She lists *Dioscorea rupicola*, *Dioscorea undatiloba* and *Dioscorea diversifolia* though not *Dioscorea multiloba*. Her paper also reports a personal communication from Codd that a preliminary investigation into the taxonomy of the three species indicated that they represented a single taxon. Simultaneously in tropical Africa, Milne-Redhead (1975) sank var. ukamensis into *Dioscorea buchananii* with two later heterotypic names, *Dioscorea mildbraediana* R.Knuth and *Dioscorea rhacodes* R.Knuth. He stated that “the plentiful material now available shows that no taxonomic value can be placed on the leaf shape and degree of lobing”. Wilkin (2001; 2009) used the same broader taxon concept as Milne-Redhead but did not consider the species in South Africa except to highlight the floral differences between *Dioscorea buchananii* and *Dioscorea rupicola*.

The taxa of the *Dioscorea buchananii* complex share perennial, subterranean tubers, left-twining habit, a tendency to possess palmately lobed leaves (but with entire leaves in some to many populations), relatively short spicate to racemose inflorescences, paired floral bracts and flowers with well-developed receptacles and seeds that are winged all round the margin but with a wing that is longer than wide (often oblong-elliptic). The comments reported above by Knuth and von Teichman suggested that there are fewer morphological entities than species currently recognised; Govaerts and Wilkin (2015) listed *Dioscorea multiloba*, *Dioscorea natalensis*, *Dioscorea rupicola*, *Dioscorea undatiloba* and *Dioscorea buchananii* as accepted species names. Thus the morphology of all the taxa listed above was studied in more detail to test this hypothesis, focussing on specimens from South Africa but including data on *Dioscorea buchananii* from throughout its geographical and morphological ranges obtained for Wilkin (2001, 2009).

## Materials and methods

The results and revised classification presented below are based on study of specimens at the following herbaria B (images), BOL, BM, K, P, PCE, PRE, NU (images) OXF, TCD and WAG and for *Dioscorea buchananii* COI, LISC, LMU, MAL and SRGH and include character data previously published in Wilkin (2001, 2009) for *Dioscorea buchananii*. The characters given in the descriptions were scored or measured using the naked eye, a dial caliper or a dissecting microscope with a graduated eyepiece. Leaf lobe lengths were made along the central vein from level with the base of the adjacent sinus to the lobe apex. The research undertaken was part of the eMonocot project and the nomenclatural and descriptive content and images form part of the Dioscoreaceae scratchpad (http://dioscoreaceae.e-monocot.org) and hence the eMonocot portal (http://e-monocot.org/).

## Results and discussion

Floral morphology indicates that there are three taxonomic entities in the *Dioscorea buchananii* species complex, not one as reported in von Teichman et al. (1975). The most easily distinguished (see Table 1) has 3 stamens in its male flowers (rather than 6), male flowers that are pendent on an erect axis via recurved 0.6-1.5 mm long pedicels and erect to ascending yellow-green tepals with cucullate (rather than flat) apices in both male and female flowers. This corresponds to the type of *Dioscorea rupicola* and is usually encountered at altitudes between 2100 and 1200 m in the Eastern Cape and KwaZulu Natal. A second taxon has patent, (sub)sessile male flowers with spreading pale green tepals in both sexes. The earliest applicable name is *Dioscorea multiloba*. The types of *Dioscorea digitaria* and *Dioscorea natalensis* are lobed and entire-leafed forms of this species respectively. It is largely allopatric with *Dioscorea rupicola*, occurring below 800m in KwaZulu Natal and rarely at higher altitudes towards the edges of its range. The final entity has patent pale green or green-yellow to purple-, pink- or bronze-hued male flowers on pedicels at least 1.7 mm long. It also differs from *Dioscorea multiloba* in its male and female tepal and torus dimensions (Table 1), albeit that female specimens with flowers at anthesis are few in number. This is *Dioscorea buchananii*, which has a wide ecological range in southern tropical Africa.

**Table 1.** The principal characters differentiating *Dioscorea rupicola*, *Dioscorea multiloba* and *Dioscorea buchananii* and their states in seven critical South African specimens. All measurements in mm.

	*Dioscorea rupicola*	*Dioscorea multiloba*	*Dioscorea buchananii*	*Junod* 1416 (K sheet)	*Junod* 2182	*Breyer* in TM23387	*Medley Wood* 11673	*Medley Wood* 12969	*Gerrard & McKen* 1617	*Pole Evans* 4854
Leaf margin	(3-) 5(-7) shallow to deep basal lobes, very rarely entire	Entire to with 3, 5 or 7 shallow to deep lobes often towards blade base	Entire to with 3, 5 or 7 shallow to rarely deep lobes towards blade base	3 or 5 basal lobes, weak secondary lobing primarily on central lobe	3, 5 or 7 basal lobes, weak secondary lobing primarily on central lobe	Deeply 5 or 7-lobed, with irregular secondary lobing especially on central lobe margins	Palmately 5 or 7-lobed with irregular secondary lobing on each lobe	Palmately 5 or 7-lobed with irregular secondary lobing on each lobe	Palmately 5 or 7-lobed with irregular secondary lobing on each lobe	3 or 5 shallow to deep basal lobes
♂ infl. length	20–86	10–88	16–70	14–42	14–32	15–34	5–12	10–33	24–28	6–21
♂ infl. habit	Erect	Pendent to spreading	Pendent to spreading	Pendent	Pendent	Pendent to spreading	Pendent	Pendent to spreading	Pendent to spreading	Pendent
♂ infl. flower organisation on axis	Solitary	Solitary or rarely in clusters of 2-3	Solitary or rarely in cymules of 2–3 flowers	Solitary or rarely in cymules of 2–3 flowers	Solitary or rarely in cymules of 2–3 flowers	Solitary or rarely in cymules of 2–3 flowers	Solitary	Solitary	Solitary	Solitary
♂ floral orientation	Pendent on axis via recurved pedicels	Patent to axis	Patent to pendent axis	Patent to pendent axis	Patent to pendent axis	Patent to pendent axis	Patent to pendent axis	Patent to pendent axis	Patent to pendent axis	Patent to pendent axis
♂ & ♀ tepals at anthesis	Erect to ascending	Spreading	Spreading	Spreading	Spreading	Spreading	Spreading	Spreading	♀ spreading, ♂ post anthesis	Spreading
♂ flowers pedicel length	0.6–1.5	(Sub)sessile	2.0–5.0	1.9–3.4	1.7–2.3	2.7–3.5	1.9–3.0	1.9–2.3	2.1–3.7	0.15–0.5
♂ Outer tepal L × W	2.0–3.8 × 0.6–1.3	1.4–2.1 × 0.7–1.5	2.5–4.7 × 1.1–2.3	2.9–3.6 × 1.9–2.1	2.3–3.3 × 1.8–2.2	2.5–3.7 × 1.3–1.8	2.7–3.7 × 1.3–1.6	2.3–3.2 × 1.4–2.0	2.5–3.0 × 1.1–1.3	1.9–2.7 × 1.6–1.9(–2.3)
♂ Inner tepal L × W	2.0–3.7 x0.8–1.5	1.2–2.2 × 0.7–1.3	2.7–4.5 × 1.3–2.3	3.0–3.7 × 1.7–2.1	2.3–3.4 × 1.4–1.7	2.9–3.2 × 1.5–1.8	2.6–3.3 × 1.0–1.8	2.5–2.9 × 1.3–1.8	2.5–3.0 × 1.1–1.3	1.9–2.9 × 1.6–2.5
♂ torus diam.	1.0–2.1	1.5–2.3	2.3–5.0	2.5–3.1	2.3–3.1	2.2–3.1	1.9–3.4	2.2–2.9	1.5–1.9 (post–anthesis/withered)	2.7–3.3
Stamen no.	3	6	6	6	6	6	6	6	Unknown	6
Filament L	0.5–1.0	0.35–0.7	0.6–1.8	1.0–1.6	0.8–1.3	0.7–0.8	1.1–1.6	0.9–1.1	N/A	0.5–0.7
Anther L × W	0.25–0.35 × 0.25–0.35	0.3–0.5 × 0.2–0.45	0.5–1 × 0.4–0.8	0.6–0.7 × 0.3–0.5	0.5–0.7 × 0.3–0.5	0.7–0.8 × 0.3–0.7	0.9–1.2 × 0.6–0.9	0.7–0.8 × 0.4–0.7	N/A	0.5–0.6 × 0.25–0.35
♀ tepal L × W	2.3–3.1 × 0.7–1.7	1.5–2.5 × 0.8–1.5	2.9–4.5 × 0.8–2.0	N/A	2.6–3.2 × ca 1.4	N/A	N/A	N/A	1.9–3.2 × 0.9–1.1	N/A
♀ Torus diam.	1.8–2.5	1.7–2.4	2.5–4.5	N/A	Ca 2.9	N/A	N/A	N/A	2.7–3.3	N/A
Capsule L × W	(18–)20–30 × 13–20	16–28 × 15–20(–22)	22–30 × (13–)18–32	N/A	N/A	N/A	N/A	N/A	N/A	N/A
Seed wing L × W	10.5–18.8 × 6.7–8.3	11–14 × 6.5–9.3	10–20 × 7–13	N/A	N/A	N/A	N/A	N/A	N/A	N/A
Seed L × W	4.6–6.5 × 5.0–6.5	4.6–5.0 × 3.7–5.0	2.5–4 × 3–4	N/A	N/A	N/A	N/A	N/A	N/A	N/A

**Figure 1.**
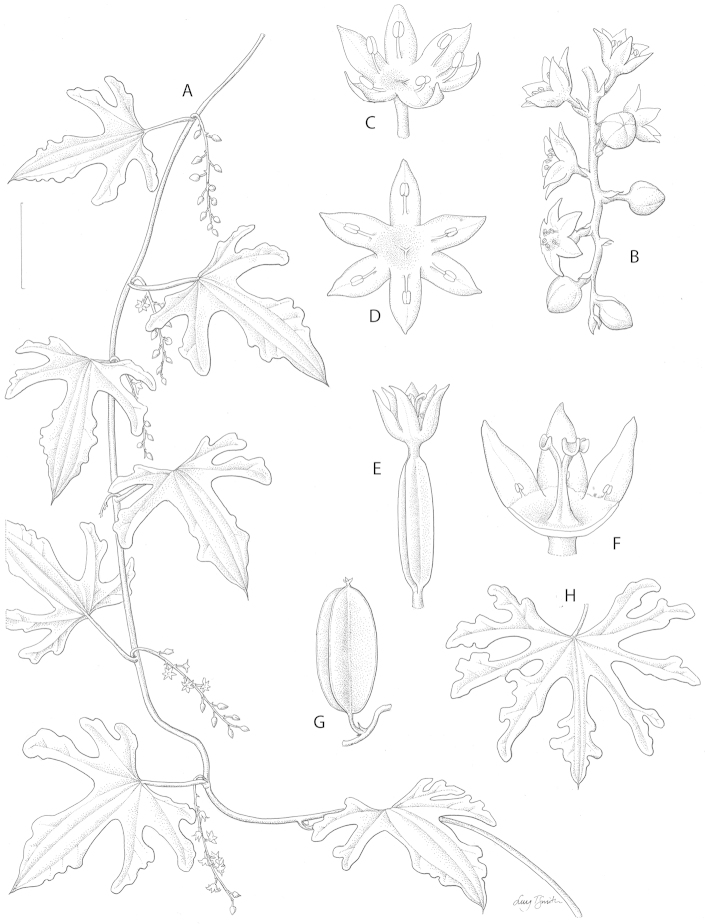
Dioscorea buchananii Benth. subsp. undatiloba vegetative and reproductive morphology **A** Habit of male plant with axillary inflorescences **B** Apical part of male inflorescence showing tepal shape and habit and bud shape **C** Rehydrated male flower with apical part of pedicel showing stamen morphology (NB tepal and stamen habit influenced by specimen preparation) **D** Fully opened out rehydrated male flower from above showing tepal shape, torus and pistillode **E** Female flower with ovary at late anthesis, tepals ascending **F** Female flower with 3 tepals removed showing torus, staminodia and gynoecium **G** Immature capsule with pedicel, floral bract and persistent tepals at apex **H** Leaf of KwaZuluNatal form of Dioscorea buchananii Benth. subsp. undatiloba. Scale bar: **A**, **H** 3 cm; **B** 7 mm; **C**, **D**, **E** 5 mm; **F** 3 mm; **G** 2 cm. From *Breyer* in TM 23387 (**A**), *Junod* 2182 (**B**, **G**), *Medley Wood* 11673 (**C**, **D**) *Gerrard & McKen* 1617 (**E**, **F**) *Medley Wood* 12969 (**H**). Drawn by Lucy Smith.

During the study, six specimens from South Africa were encountered that possessed inflorescence and floral morphology similar to that of *Dioscorea buchananii* but whose leaves had at least a degree of secondary (pinnatifid) marginal lobing, especially on the central primary lobe (Figure 1A, H). This has not previously been recorded for the species. Milne-Redhead (1975) showed that both entire and palmately lobed leaves were found in *Dioscorea buchananii* and the collections made since 1975 confirm this hypothesis and support reducing Dioscorea buchananii var. ukamensis to synonymy. The secondary lobing, in combination with the observation that inflorescence and floral dimensions in the six specimens overlap with those of *Dioscorea buchananii* (Table 1), but are in the lower part of its ranges of variation, suggest that South Africa has a distinct subspecies of *Dioscorea buchananii* that corresponds with the types of both *Dioscorea undatiloba* and *Dioscorea junodii*. The former is the earlier name so Dioscorea buchananii subsp. undatiloba (Baker) Wilkin is applicable. A further specimen, *Pole Evans* 4954 collected from Ixopo in KwaZulu Natal but flowered in cultivation, was tentatively linked to *Dioscorea multiloba* due to the absence of secondary leaf lobing, a pendent inflorescence, flowers with spreading tepals and incurved filaments over three depressions in the centre of the torus. However, the presence of short pedicels and the tepal and torus dimensions suggest potential introgression with Dioscorea buchananii subsp. undatiloba. The two taxa are sympatric in KwaZulu Natal. In both this and the case above, an extensively sampled population-based study is needed using molecular marker data.

## Taxonomy

### Key to male flowering plants of the *Dioscorea buchananii* Benth species complex

**Table:** 

1	Stamens 6, inflorescences spreading to pendent, tepals spreading, apices flat	**2**
–	Stamens 3, inflorescences erect, and bearing flowers patent to axis to recurved towards its base, tepals erect to ascending, apices cucullate	***Dioscorea rupicola***
2	Flowers subsessile, tepals 1.2–2.2 m long, buds (sub)globose	***Dioscorea multiloba***
–	Flowers on 1.7–5.0 mm long pedicels, tepals 2.3–4.7 mm long, buds turbinate	**3**
3	Leaves entire to moderately lobed at stem base and on vegetative stems but then less strongly lobed to entire on reproductive shoots, lobe margins at most weakly undulate	**Dioscorea buchananii subsp. buchananii**
–	Leaves consistently moderately to deeply 3, 5 or 7-lobed from stem bases to apices, with irregular, pinnate secondary lobing present on central lobe at least	**Dioscorea buchananii subsp. undatiloba**

### Key to female plants of the *Dioscorea buchananii* Benth species complex

**Table:** 

1	Floral torus diameter 2.5–4.5 mm. Capsule oblong to obovate to very broadly so or rotund in outline, capsule width (13–)18–32 mm	**2**
–	Floral torus diameter 1.7–2.5 mm. Capsule oblong to narrowly obovate in outline, capsule width 13–20(–22) mm	**3**
2	Leaves entire to moderately lobed at stem base and on vegetative stems but then less strongly lobed to entire on reproductive shoots, lobe margins at most weakly undulate	**Dioscorea buchananii subsp. buchananii**
–	Leaves consistently moderately to deeply 3, 5 or 7-lobed from stem bases to apices, with irregular, pinnate secondary lobing present on central lobe at least	**Dioscorea buchananii subsp. undatiloba**
3	Tepals erect, staminodia 3	***Dioscorea rupicola***
–	Tepals spreading, staminodia 6	***Dioscorea multiloba***

#### Dioscorea buchananii

Taxon classificationPlantaeDioscorealesDioscoreaceae

Benth.

Dioscorea buchananii Benth., Hooker’s Icon. Pl. 14:76, t. 1397, 1398 (1882).
Dioscorea buchananii var. ukamensis R.Knuth in H.G.A Engler (ed.), Pflanzenr. 4, 43: 185 (1924). Type: Tanzania, Morogoro Dist., Ukami, without date, *Stuhlmann* 8283 (holotype: B†).
Dioscorea mildbraediana R.Knuth, Notizbl. Bot. Gart. Berlin-Dahlem 11: 1059 (1934).
Dioscorea buchananii Type: Tanzania, Kilwa, Mswega, ♂ fl. 22 Jun 1932, *Schlieben* 2495 (holotype: B!; isotype BR!).
Dioscorea rhacodes Peter ex R.Knuth, Repert. Spec. Nov. Regni Veg. 42: 162 (1937).
Dioscorea buchananii Type: Tanzania, Ukami, east of Morogoro, ♂ fl. 1926, *Peter* 46419 (holotype: B!).

##### Types.

Malawi: Shire Highlands, ♂ fl. 1881, *Buchanan* 173 (syntype: K!) ♀ fr. 1881, *Buchanan* 358 (syntype: K!).

##### Description.

Twining vine to 10 m in height, vegetative growth annual, usually 1 shoot per year from apex of perennial, woody tuber, to ca 20 cm in diam., usually globose to ovoid, sometimes elongate or irregular, shape varying perhaps based on rockiness of substrate, externally dark grey to brown, fissured, bark-like. Indumentum absent. *Stems* left-twining, to 5 mm in diam., terete to shallowly longitudinally ridged, more so when dry, unarmed from base, green or purple-hued, herbaceous, cataphylls not seen. *Leaves* alternate, blade variable, 2.8–13.5 × 1.1–14.7 cm, entire or with 3, 5 or 7 shallow to deep lobes, ovate to broadly so, veins 7(–9), primary venation in shallow channels on upper surface in fresh material, primary and secondary venation prominent below, base cordate, with a shallow to deep basal sinus, rarely truncate, texture chartaceous, where lobes present central lobe to 116 mm long, lateral lobes to 35 mm long, lobes usually found primarily in vegetative stem leaves with reproductive stem leaves more weakly lobed to entire, rarely consistently lobed to shoot apices, lobes inserted from around mid-point to point of petiole insertion, lobe margins entire to (rarely) weakly undulate or in some leaves lobed to stem apices with weak to strong irregularly pinnate secondary lobing, blade or central lobe apex acute to triangularly short-acuminate, rarely obtuse or truncate, bearing a 1.5–10 mm long, thickened, very narrowly triangular, brown forerunner tip fed by the 3 central veins of blade; petiole 0.6–6.3 cm long, ridged like stem and with a narrow channel on upper surface, colour as stem, pulvinii sometimes paler or purple-hued; lateral nodal organs absent but petiole base broader where inserted onto stem, axillary bulbils absent. Inflorescences simple, usually 1 per axil, axes straight, angular, pale green or purple or brown-hued; male 1.6–7 cm long, peduncle 2–11 mm long, racemose, pendent to spreading, usually dense with flowers 0.3–4.1 mm apart and solitary or rarely in cymules of 2–3 flowers, on a 1.7–5.0 mm long pedicel that is angular and slightly broader towards apex, buds patent to axis, pendent at developing inflorescence apex only in very early development; female inflorescence 9–77 mm long, accrescent to ca 30 cm long in fruit, peduncle 12–20 mm long, spicate, pendent, lax, flowers subpendent only at the earliest stages of development, patent to axis at anthesis but ascending to erect soon thereafter. Flowers turbinate in bud, tepals 6, free, inserted on margin of a saucer-shaped, weakly thickened torus, spreading at anthesis, sometimes ascending thereafter, whorls scarcely differentiated, 3–veined, brown, green, olive or bronze, sometimes with a pink or yellow hue or mottled; male flower with floral bract and bracteole sheathing pedicel base, bract 1.6–2.6 mm long, ovate, long-acuminate, membranous, bracteole similar, narrower, usually offset from bract; outer tepals 2.5–4.7 × 1.1–2.3 mm, inner tepals 2.7–4.5 × 1.3–2.3 mm, narrowly ovate to lanceolate or triangular, chartaceous, apex acute to short–acuminate, flat; filaments 0.6–1.8 mm long, erect but incurved over 2.3–5 mm diam. torus, anthers 0.5–1.2 × 0.4–0.8 mm, introrse; pistillode to ca 0.1 mm high, 3 centrally fused triangular ridges at 120° to each other in flat central part of concave torus; female flower with floral bract and bracteole sheathing ovary base, bract 1.6–2.4 mm long, ovate, long-acuminate, membranous, bracteole similar, narrower, usually offset from bract; ovary 5–10 mm long, 3-angled, lorate to very narrowly elliptic in outline, colour as axis, apex constricted; outer tepals 2.9–4.5 × 0.8–1.9 mm, inner tepals 2.9–4.4 × 0.9–2 mm, more or less erect, narrowly ovate to lanceolate, apex acute to short-acuminate, flat, each tepal with 0.2–0.7 mm long basal staminode inserted at the boundary with the torus at the tepal base midpoint, usually fleshy and ovoid but sometimes substaminiform; torus 2.5–4.5 mm in diam. both tepals and torus accrescent as ovary enlarges; style 1.8–3.2 mm long, erect, divided into 3 spreading branches towards apex, stigmas bifid, oblong to clavate. Capsule 2.2–3 × (1.8–)2–3.2 cm, pedicel reflexed and thus ascending to erect at dehiscence, lobes obovate to oblong-obovate in outline, thick-chartaceous, base and apex usually truncate, dry and withered flowers persistent until relatively late in development on a ca 1.2–2.0 mm long stipe, light brown with chestnut-brown to coppery brown mottling, dehiscing apically at least at first. Seed 2.5–4 × 3–4 mm, irregularly lenticular, dark brown wing 1–2 × 0.7–1.3 cm, broadly oblong-elliptic to rotund to irregularly so, wing extending all around seed margin although elongated towards rounded to obtuse base and apex, pale brown, translucent with fine paler speckling.

#### Dioscorea buchananii subsp. buchananii

Taxon classificationPlantaeDioscorealesDioscoreaceae

##### Description.

Leaves entire to moderately 3, 5 or 7-lobed at stem base and on vegetative stems but then less strongly lobed to entire on reproductive shoots, lobe margins at most weakly undulate; where lobed central lobe usually the largest, maximum length as in species as a whole, broadly ovate to lanceolate or deltoid, lateral lobes oblong to rounded. Male flower pedicel, tepal and torus dimensions as in species as a whole.

##### Distribution.

Tanzania and Southern and Eastern Congo (Kinshasa) to southern Mozambique, Zimbabwe and Angola.

##### Vernacular name(s).

See Wilkin (2001, 2009).

##### Ecology.

Frequently associated with rocky habitats, often in *Brachystegia* woodland, but also on termitaria, in riverine forest and near mangrove swamps, on limestone and granite substrates; sea level to 1600 m (Wilkin 2001, 2009).

##### Conservation.

The broad southeastern African distribution of this species indicates that its EOO and AOO will greatly exceed the threshold for threatened IUCN categories (20, 000 km2/2000 km2) (IUCN 2001) and its provisional status is LC.

##### Uses.

None known.

##### Specimens examined.

Representative specimens are cited in Milne-Redhead (1975) and Wilkin (2001, 2009).

#### Dioscorea buchananii subsp. undatiloba

Taxon classificationPlantaeDioscorealesDioscoreaceae

(Baker) Wilkin comb. & stat. nov.

urn:lsid:ipni.org:names:77146550-1

Figure 1

Dioscorea undatiloba Baker, J. Bot. 27: 8 (1889); R.Knuth in H.G.A Engler (ed.), Pflanzenr. 4, 43: 184 (1924).
Dioscorea buchananii subsp. undatiloba Type: South Africa. KwaZulu Natal: Port Natal, Mandini District, Tugela, ♂ fl. & ♀ fl. without date, *Gerrard & McKen* 1617 (holotype: K post anthesis ♂ fl. & ♀ fl. [K000098906!]; isotype: TCD!, ♀ fl.!)
Dioscorea junodii Burtt-Davy, Kew Bull. 1924: 231 (1924), **synon. nov.**
Dioscorea buchananii subsp. undatiloba Type: South Africa. Limpopo: Mopani District, Valley of Schambock’s Stadt, near Shilouvane, (Shiluvane) Sanatorium, 24°02'20"S, 30°16'59"E, ♂ fl. *Junod* 1416 (holotype: K [K00098905!]), *non* PRE [PRE0093186-0, digital image!]

##### Type.

Based on *Dioscorea undatiloba* Baker.

##### Description.

Leaves consistently moderately to deeply 3, 5 or 7-lobed from stem bases to apices, with irregular, pinnate secondary lobing present on central lobe at least; central lobe to 50 mm long, lanceolate to elliptic or rhomboid, lateral lobes to 33 mm long, oblong to narrowly so, central lobe largest to lobes more or less equal in length and width. Male flowers on 1.7–3.7 mm long pedicels, tepals 2.3–3.7 × 1.0–2.2 mm, torus (1.5–)1.9–3.4 mm in diam.

##### Distribution.

Endemic to South Africa in Limpopo and KwaZulu Natal provinces. The Limpopo specimens are from localities relatively close to those of the type subspecies in Gaza province of Mozambique.

##### Vernacular name(s).

Not known.

##### Ecology.

*Ca.* 50 to 600 m altitude in KwaZulu Natal, and 700 to 1000 m in Limpopo. Associated geology, soils and vegetation unknown.

##### Conservation.

Dioscorea buchananii subsp. undatiloba has not been recorded since 1921 in either Limpopo or KwaZulu Natal and is known from only six specimens. However, pending urgently needed searches to find extant populations in Limpopo, KwaZulu Natal and the intervening areas and further research on the relationships between those populations, the most appropriate provisional conservation status assessment is DD. It is conceivable that this taxon is already extinct in part or all of its range, especially extensively developed lowland KwaZulu Natal.

##### Uses.

None known

##### Notes.

The specimen labelled *Junod* 1416 at PRE [PRE0093186-0] is male flowering material of *Dioscorea sylvatica* Ecklon. This explains the placement of *Dioscorea junodii* in the synonymy of that species in Govaerts and Wilkin (2015). However, Burtt Davy would have examined and described the K sheet cited above, hence its holotype status and synonymy under Dioscorea buchananii subsp. undatiloba. No specimen of *Junod* 1416 appears to be preserved at G or Z based on their online databases.

##### Specimens examined.

**South Africa. Limpopo:** Mopani District, Shiluvane, 24°02'20"S, 30°16'59"E, ♂ fl. & ♀ fr. May 1905, *Junod* 2182 (Transvaal Museum 7164), (PRE!); Louis Trichardt, ♂ fl. Jan 1921, *Breyer in TM* (Transvaal Museum) 23387 (K!, PRE). **KwaZulu Natal:** uThungulu District, Umvuzaan (uMvuzane) Valley, 28°46'21"S, 31°20'29"E, ♂ fl. buds Jan 1915, *Medley Wood* 12969 (K!); Pietermaritzburg, Camperdown 29°43'59.91"S, 30°31'59.87"E ♂ fl. Mar 1910, *Medley Wood* 11673 (K!).

#### Dioscorea multiloba

Taxon classificationPlantaeDioscorealesDioscoreaceae

Kunth

Figure 2 , 3A, B

Dioscorea multiloba Kunth, Enum. Pl. 5: 376 (1850); R.Knuth in H.G.A Engler (Ed.), Pflanzenr. 4, 43: 183 (1924).
Dioscorea diversifolia Kunth, Enum. Pl. 5: 375 (1850), *non* Griseb. in C.F.P.von Martius (Ed.), Fl. Bras. 3(1): 41 (1842), type only.
Dioscorea multiloba Type: South Africa. Eastern Cape: Pondoland, between Umtentu and Umzimkulu Rivers, ♂fl. & ♀ fr., Feb, year unknown, *Drège* 4497 (holotype: B [B_10_0278736 ♂fl. digital image!]; isotypes: G [G00018717 ♂fl. digital image!]; K [K000098903,K00098904 ♂fl. & ♀old fr.!]; OXF [OXF0004195 ♂fl. & ♀ old fr.!]; P [P00440190! ♀ fr.!]; TCD ♂fl.!).
Dioscorea natalensis R.Knuth in H.G.A Engler (ed.), Pflanzenr. 4, 43: 94 (1924), **synon. nov.** Type: South Africa. KwaZulu Natal: Durban [Kingsburgh], Winkle Spruit, ♂fl. 28 Feb 1912, *Rudatis* 1609 (holotype: B [B_10_0160974 digital image!]; isotypes K! [K000815875, K000815874] WAG [WAG0027134!]; Z [Z-000065671, Z-000065672, digital images!].
Dioscorea digitaria R.Knuth in H.G.A Engler (ed.), Pflanzenr. 4, 43: 184 (1924). Type: South Africa. KwaZulu Natal Friedenau, Umgayeflat (Alexandria), ♂fl. 1 Oct 1909, *Rudatis* 724 (holotype: B [B_10_0160991 digital image!]).

##### Type.

South Africa. KwaZulu Natal: “Probably collected in Pondoland”, ♂ fl. & ♀ fr. 1840, *Drège* 4496 (lectotype: K! [K000098902 ♂fl. buds & ♀ fr.]; isolectotypes B [B_10_0004142 ♂fl. buds & ♀ fr., B_10_0004143 ♂fl. buds digital images!]; G [G00018675♀ fr. digital image!]; K [K000098900 ♂fl. buds & ♀ fr.!]; KIEL; OXF! [OXF00004193♀ fr.]; P [P00440187 ♀ fr., P00440188 ♂fl. buds & ♀ fr., P00440189 ♀ fr., P00440191 ♂fl. buds & ♀ fr.!], TCD ♀ fr.!).

##### Description.

Twining vine to ca 3 m in height, vegetative growth annual from a perennial tuber. Tuber apex only seen, ca 15 cm in diam., convex, dark brown to black, bark-like, bearing one shoot per year at central apex, according to von Teichman und Logischen et al. (1975) lobed and irregular below ground but not branched like that of *Dioscorea rupicola*. Indumentum absent. Stems left-twining, to ca 5 mm in diam., terete to shallowly longitudinally ridged and more so when dry, base with dense, firm processes to ca 1 mm long, dark purple-brown, becoming unarmed above and pale green to dull purple-hued. Cataphylls present towards stem base, ovate, acuminate, concolorous with stem to paler. Leaves alternate, blade 15–148 × 8–133 cm, ovate to narrowly or broadly so in outline, deeply to shallowly 3- to 7-lobed, usually lobed at base at least, blade sometimes (sub)entire in especially in leaves on terminal shoots, lobing usually concentrated in basal part of leaf close to point of petiole insertion, texture thinly to thickly chartaceous, primary venation in shallow channels on upper surface in fresh material sometimes bullate between secondary veins, primary and secondary venation prominent below, dark to mid green above, pale below, base cordate to truncate, sinus where present to 31 mm deep, lobes to 42 mm long, apically obtuse to rounded, lobe margins entire to sometimes with weak secondary lobing, apical lobe lanceolate-deltoid to broadly ovate, apex acute to obtuse, bearing a thickened, narrow, caudate forerunner tip to 7 mm long, sometimes subterminal, derived from central 3 veins of blade, pale yellow-green when fresh, brown and with margins curled inwards on upper surface when dry, primary veins 5–9, 3 in apical lobe, usually 1 per basal lobe but sometimes multiple veins per lobe in 3-lobed or entire leaves; petiole 6–54 mm long, ridged and with a narrow channel on upper surface, colour as stem, upper pulvinus sometimes paler than lower; lateral nodal organs absent and nodes not thickened but in vegetative stem leaves petiole base broader where inserted onto stem; axillary bulbils not present. Inflorescences simple, usually spicate, axes angular, pale green; male inflorescences 1-6 per axil, often 1, sometimes borne on weak axillary shoots with few to no leaves, 10–88 mm long, peduncle 2–8 mm long pendent to spreading, never erect, axis usually straight but sometimes irregularly flexuous, flowers usually solitary or rarely in clusters of 2–3, 1.2–4.6 mm apart, buds patent to axis, pendent at developing inflorescence apex only in very early development; female inflorescences 7–84 mm long, peduncle 4–10 mm long, flowers patent to axis at anthesis but ascending to erect soon thereafter. Flowers (sub)globose in bud, tepals 6, free, inserted on a saucer-shaped torus, spreading at anthesis, whorls scarcely differentiated, yellow-green to pale green, often with a darker apical mark or darker on midrib; male (sub)sessile, floral bract 1.0–1.7 × 0.4–0.6 mm, ovate to lanceolate, acuminate, concolorous with axis, bracteole similar, narrower, usually offset from bract; outer tepals 1.4–2.1 × 0.7–1.5 mm, inner 1.2–2.2 × 0.7–1.3 mm, ovate to narrowly so or deltoid, apex acute to obtuse, slightly thickened and sometimes with upcurved margins but not cucullate, inserted on margin of 1.5–2.3 mm diam. torus, thicker than tepals (but less so than in *Dioscorea rupicola*) and concolorous with them, stamens 6, inserted at torus/tepal boundary at tepal base midpoint, filaments 0.35–0.7 mm long, erect but markedly incurved such that anthers are held over concave surface of torus with apices sometimes almost touching, anthers 0.3–0.5 × 0.2–0.45 mm, oblong to oblong-elliptic, basifixed, pale yellow, pistillode 0.1–0.7 mm long, variable in shape but formed by 3 centrally fused triangular ridges at 120° to each other, with a 0.35–0.6 mm diam. bowl-shaped, concave, circular to ovoid in outline, possibly nectariferous depression between each lobe demarcated by a membrane and with a denser texture than torus, pistillode apex either acute or bearing short recurved lobes; female flowers sessile, floral bract 0.9–1.6 mm long, appressed to ovary base, otherwise bract and bracteole as male; ovary 3.0–6.1 mm long, 3-angled, lorate to very narrowly elliptic in outline, pale green, apex constricted, tepals 1.5–2.5 × 0.8–1.5 mm, shape, apex and colour as male, torus 1.7–2.4 mm in diam., Tepals and torus accrescent as ovary enlarges when flowers persist and tepals sometimes becoming ascending but not erect, staminodia 6, ca 0.1 mm long, inserted at torus/tepal boundary at tepal base midpoint, style ca 1.0 mm long, erect, stout, 3-angled, broadest at base, stigmas 3, ca 0.4 × 0.9 mm, spreading, bifid, lobes broadly ovate in outline. Capsule 16–28 × 15–20(–22) mm, pedicel reflexed and thus ascending to erect at dehiscence, oblong to obovate in outline, thick-chartaceous, base obtuse to truncate, apex truncate to rounded, dry and withered flowers persistent until relatively late in development on a ca 1.0–1.7 mm long stipe, pale brown with darker coppery-brown speckling, dehiscing apically at least at first. Seed 4.6–5.0 × 3.7–5.0 mm excluding wing, lenticular, dark brown, wing 11–14 × 6.5–9.3 mm, oblong to irregularly elliptic, all round margin though with elongated towards rounded to obtuse base and apex, pale brown, translucent with fine paler speckling.

**Figure 2.**
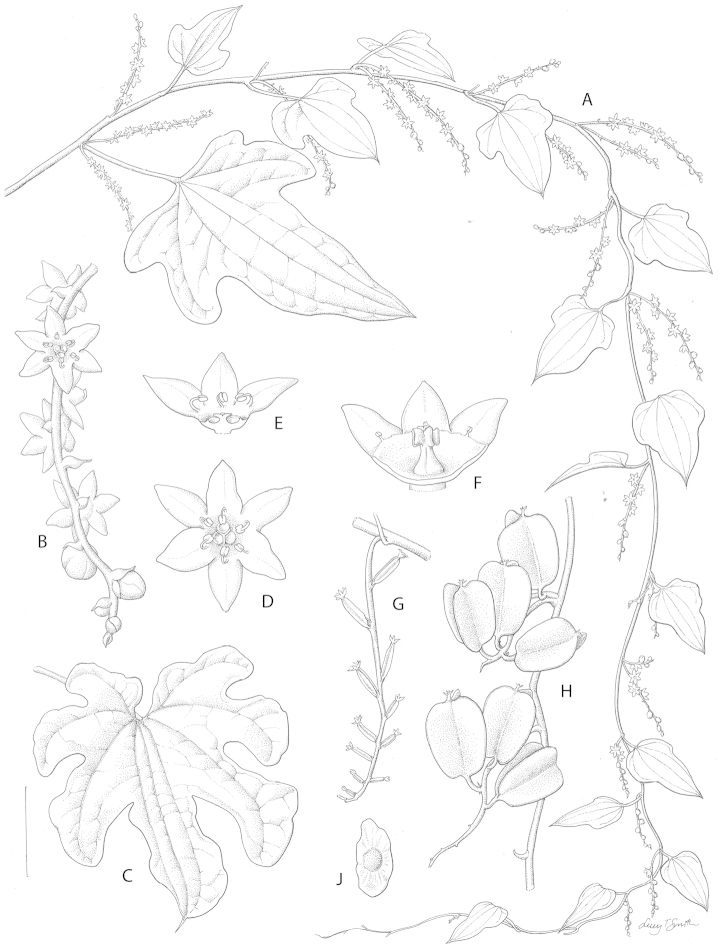
*Dioscorea multiloba* Kunth vegetative and reproductive morphology. **A** Habit of male plant with axillary inflorescences **B** Apical part of male inflorescence showing tepal shape and habit and bud shape habit **C** Lobed leaf showing venation of upper surface and forerunner tip **D** Male flower from above showing filament habit **E** Male flower with 3 tepals and part of torus removed showing torus shape, pistillode and associated concavities in torus surface **F** Female flower with 3 tepals and part of torus removed showing torus shape, staminodia and gynoecium **G** Female inflorescence showing tepal habit and ovaries **H** Infructescence showing some submature capsules with persistent tepals **J** Seed showing wing shape. Scale bar: **A**, **C** 3 cm; **B** 7 mm; **D**, **E** 4 mm; **F** 3 mm; **G**, **H** 2.5 cm; **J** 2 cm. From *Ward* 3076 (**A**, **B**, **D**, **E**), *Medley Wood* 329 (**F**, **G**), *Gueinzius* s.n. (**H**, **J**) and a photograph (**C**). Drawn by Lucy Smith.

**Figure 3.**
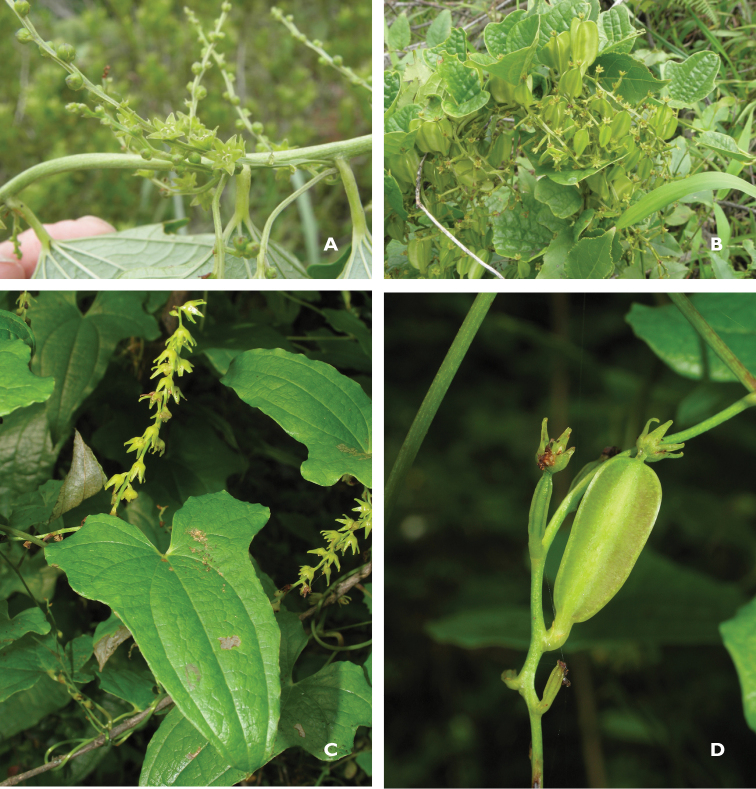
*Dioscorea multiloba* and *Dioscorea rupicola* colour photographs. **A** Upside down shoot of *Dioscorea multiloba* with male flowers **B** Female plant of *Dioscorea multiloba* with female flowers and immature capsules **C** Leaf and erect male inflorescence of *Dioscorea rupicola* showing habit of flowers. **D** Immature capsule of *Dioscorea rupicola* with persistent, erect, cucullate tepals at its apex. Photo **A**, **B:** Tony Abbott; **C**, **D:** Neil Crouch.

##### Distribution.

Endemic to South Africa (Eastern Cape to Mpumalanga) and Swaziland.

##### Vernacular name(s).

The only vernacular name known is wild yam.

##### Ecology.

*Dioscorea multiloba* occurs in a range of habitats but is principally associated with forest and bush margins and associated grasslands on a range of sandy and loamy substrates. In the northern part of its range it often appears to occur in swampy habitat. It is found at altitudes from close to sea level to 800 m in KwaZulu Natal. At the edges of its range the specimen from Swaziland (*Compton* 26691) was collected at ca 4000’ (1200 m) and *Flanagan* 2717 from the Eastern Cape 4500’ (1400 m).

##### Conservation.

*Dioscorea multiloba* is widespread in lowland KwaZulu Natal, and its distribution extends into the Eastern Cape, Mpumalanga and Swaziland. Thus its EOO and AOO will greatly exceed the threshold for threatened IUCN categories (20, 000 km2/2000 km2) (IUCN 2001) and its provisional status is LC. This status is also given by the SANBI red list programme (under *Dioscorea diversifolia* Griseb.)

##### Uses.

None known. Data on the steroid content of this species is desirable. Codd (1960) states that only three South African species contain diosgenin but the data on which this is based are not presented.

##### Notes.

Kunth cites male and female syntypes as follows “Drège, Herb. Cap. no. 4495. ex parte. v.s. in Herb. Luc. and Drège, Herb. Cap. No 4496. ex parte. v.s in Herb. Luc.” Herb. Luc. appears to be an abbreviation for Herbarium Lucae, which formed part of the KIEL herbarium. Kunth specified that 4495 was male and 4496 female. However, there is both male and female material at K under 4496. In contrast, a single sheet in B (B_10_0004142) has fragments of both male and female plants under both collection numbers. P has female material under 4495 (P00440190) and male material under 4496 (P00440188) among 5 duplicates of both numbers. Given this confusion and the number of duplicates in European herbaria it was decided to lectotypify the species using K000098902, the most complete and representative specimen available.

The only specimen cited in the protologue of *Dioscorea diversifolia* Kunth, a later homonym of *Dioscorea diversifolia* Griseb., was *Drège* 4497. The material under this number at TCD appears to represent two different male plants of *Dioscorea multiloba*, with the left and bottom fragments possessing flexuous inflorescences and the right a straight inflorescence. The K and OXF material is different, with entire leaves, male flowers in bud and the previous season’s fruit. K has 2 very similar sheets from both Hooker and Bentham’s herbaria.

##### Specimens examined.

**South Africa. Eastern Cape:** Komga, near Kei Mouth, ♂ fl. Jan 1890, *Flanagan* 442 (K!, PRE); Engcoba (Ngcobo) Mountain, ♀ immature fr. Jan 1896, *Flanagan* 2717 (PRE!); Mquanduli, Coffee Bay, ♂ fl. 4 Mar 1953, *Theron* 1505 (PRE!); Pondoland, between Umtentu River and Umzinkulu River, ♂ fl.buds & old fr. Feb unknown year, *Drège* 4497 (K!, OXF!, TCD!). **KwaZulu Natal:** Port Shepstone, Mgayi, 30°25'S, 30°30'E, ♂ fl. 27 Jan 1968, *Ward* 6339 (NU [NU0028305], UDW); Port Shepstone, Mgayi, “30°25'S, 30°25'E”, ♀ immature fr. 27 Jan 1968, *Ward* 6340 (K!, NU [NU0028304 digital image!], PRE, UDW digital image!); Inanda, ♀ fl. & immature fr. Jan ?1879, *Medley Wood* 329 (K, 2 sheets!); Port Shepstone, Umtamvuna Nature Reserve, Beacon Hill, 31°00'33"S, 30°10'55"E, ♀ fl. & immature fr. 9 Dec 2010, *Abbott* 9287 (PCE!, PRU!); Port Shepstone, Umtamvuna Nature Reserve, Beacon Hill, 31°00'33"S, 30°10'55"E, ♂ fl. 9 Dec 2010, *Abbott* 9288 (PCE!, PRU!); Inanda, ♂ fl. Jan. ?1880, *Medley Wood* 825 (K!); Inanda, Groenberg, ♂ fl. Mar ?1880, *Medley Wood* 892 (K!); Lions River District, collected at Karkloof 11 July 1952 and cultivated at Irene, ♀ immature fr. 20 Dec 1954, *Pole-Evans* 4862 (K!, PRE); Durban, Umbilo Waterfall, ♂ fl. received Feb 1883 *Rehmann* 8155 (K!); Port Natal, ♂ fl. & ♀ fr. without date, *Gueinzius* s.n. (K!; TCD!); Port Natal, ?Umgena, ♂ fl. without date, *Gerrard & McKen* 1920 (TCD!); Fort Bowker, ♂ fl. & ♀ fr. without date, *Bowker* 575 (TCD!); Kaffraria, Tsomo (?), ♂ fl.. without date, *Bowker* 861 (TCD!); Fort Bowker ♂ fl. without date, Bowker 627 (TCD!); Natal, no further data, ♂ fl. without date, *Gerrard & McKen* 36 (TCD!); Port Natal, ♂ fl. without date, *Sanderson* 5 (TCD!); Durban District, Umbogintwini, above lagoon, st. 10 May 1964, *Ward* 4976 (NU [NU0028355 digital image!]); Pietermaritzburg District, Isipingo Flats, 29°59'S, 30°56'E ♂ fl. 1 May 1971, *Ward* 6992 (NU [NU0028390 digital image!], UDW digital image!); Lower Umfolozi District, Hluhluwe Game Reserve, ♂ fl. 24 Jan 1949, *Ward* 665 (NU [NU0028255, 0028351 digital image!]); Hlabisa District, Hluhluwe Game Reserve, ♂ fl. 16 Jan 1954, *Ward* 2073 (K!, NU [NU0028352 digital image!], PRE); Hlabisa District, Hluhluwe Game Reserve, ♂ fl. 6 Mar 1957, *Ward* 3076 (K!, (NU [NU0028353, 0028354 digital images!], PRE); Mtubatuba District, Hlabisa, Park Ridge Farm, ♀ fr. 11 May 1968, *Harrison* 498 (PRE!); Maputaland, Kosi Bay area near Catholic Mission, sterile 20 Mar 1965, *Vahrmeijer* 482 (K!, PRE); Maputaland, 3 miles from Maputa on road to Olibotini, ♂ fl.23 Mar 1965, *Vahrmeijer* 540 (K!, PRE); KZN, No further data, ♂ fl. received Jul 1965, *Gerrard* 772 (K!); ♂ fl. received Jul 1965 & Mar 1872, *Gerrard* 1920 (K!); KZN, Locality illegible, ♂ fl. 1862, Cooper 3244 (K!). **Mpumalanga:** Pilgrims Rest District, collected at Mac Mac 19 Aug 1952 and cultivated at Irene, ♂ fl. 20 Dec 1954, *Pole-Evans* 4846 (K!, 2 sheets, PRE); Pilgrims Rest District, collected at Mac Mac and cultivated at Irene, ♂ fl. 14 Apr 1955, *Pole-Evans* 4861 (K!, 2 sheets, PRE). **Swaziland:** Mankaina District, ♂ fl. 3 Feb 1958, *Compton* 27492 (K!, PRE); Mbabane District, Ukutula, ♂ fl. 21 Feb 1957, *Compton* 26691 (K!, PRE).

#### Dioscorea rupicola

Taxon classificationPlantaeDioscorealesDioscoreaceae

Kunth

Figure 3C, D

Dioscorea rupicola Kunth, Enum. Pl. 5: 378 (1850).

##### Type.

SOUTH AFRICA. No further data, plant obtained by Ecklon possibly from the Winterberg mountains cultivated in Berlin s.n., ♂ fl. 27 Jul 1836 (holotype: B†; isotype K! [K000098907!]).

##### Description.

Twining vine to not more than 5 m in height. Vegetative growth annual from a perennial tuber. Mature tuber apex to 4 × 4 cm, buried to 15 cm below soil surface, irregularly ridged longitudinally, corky, with an apical depression bearing shoot(s, usually 1 per year) and hard, brown, deltoid cataphylls to 15 mm long; base of tuber bearing 1–3 branches to 30 × 1– 3 cm long, corky and fissured externally and bearing wiry roots, parenchyma white, brittle (*fide* Archibald 1968). Indumentum absent. Stems to 4 mm in diam., left-twining, terete but longitudinally ridged, unarmed, pale green, sometimes pink, purple or brown-hued, branched above, cataphylls present towards base, to 6 × 3 mm, deltoid, apex caudate, recurved (*fide* Archibald 1968). Leaves alternate, blade 20–104 × 15–85 mm, ovate to narrowly so or lanceolate in outline, weakly to strongly 3- to 7-lobed around point of petiole insertion, rarely entire, juvenile plants with wholly entire leaves, usually broadly ovate to orbicular, texture chartaceous (thinner in juveniles), primary venation in shallow channels on upper surface in fresh material and lamina between secondary veins weakly bullate, shiny mid green above, paler below, drying olive green below, browner above; margins weakly undulate in fresh material, base cordate, sinus (1–)3–33 mm deep, lobes to 36 mm long, apically obtuse to rounded, apical lobe lanceolate to lanceolate-deltoid or lanceolate oblong, apex broadly acuminate with a 0.5–5 mm long narrow, caudate, thickened forerunner tip that appears channelled above, brown, veins usually 7, 3 in apical lobe with 2 per side running into basal lobe(s), sometimes a smaller vein close to the basal sinus, primary and secondary venation prominent below; petiole 10–67 mm long, ridged and with a narrow channel on upper surface, colour as stem, basal pulvinus flattened and broadly deltoid towards point of insertion onto node, especially in larger leaves, lateral nodal organs absent but in largest stems nodes swollen with a blunt projection on either side of petiole insertion onto node; axillary bulbils not present. Inflorescences 1 per axil, simple, axes angular, pale green, flowers campanulate; male inflorescences 20–86 mm long, peduncle 5–14 mm long, ca 1 mm in diam. at base, racemose, erect and bearing flowers ca 2–8 mm apart, buds oriented towards apex in very early development but at least patent to axis and usually recurved towards its base at anthesis, female inflorescences 9–82 mm long, peduncle 9–24 mm long, spicate, pendent, flowers oriented towards apex in very early development but usually patent to axis to ascending at anthesis. Flowers with 6 tepals, buds turbinate, apex acute-conical, at anthesis pedicel, tepals and torus exterior pale green to yellow-green, torus inner surface light pink to purple, possessing a light, sweet fragrance (*fide Moll* 1400, Archibald 1968); male flowers borne on 0.6–1.5 mm long curved, stout, obconic pedicels, floral bract 1 per flower, at pedicel base 1.0–1.7 × 0.4–0.7 mm long, ovate to narrowly so, acuminate, concolorous with pedicel and flower when fresh, paler brown than flower when dry; bracteole 1 per flower, similar, usually narrower and slightly shorter; tepal whorls virtually undifferentiated, inner slightly broader, tepals free, outer whorl 2.0–2.8 × 0.6–1.3 mm, inner whorl 2.0–3.7 × 0.8–1.5 erect to ascending, lanceolate to deltoid-lanceolate, apex acute but cucullate and appearing blunt, tepals inserted on the margin of a 1.0–2.1 mm diam. fleshy torus, when fresh externally broadly convex, internally with 3 swollen lobes forming an annulus with a central depression in flower centre, shape lost in drying but darker than tepals; stamens 3; filaments 0.35–0.7 mm long, inserted at base of each torus lobe on outer edge adjacent to outer whorl tepals, erect, weakly incurved, pale green, anthers 0.25–0.35 × 0.25–0.35 mm, very broadly oblong-orbicular, introrse, basifixed, pale yellow; pistillode ca 0.1 mm long, conical; female flowers overall shape as male, sessile, floral bract 1.0–2.0 × 0.6–1.2 mm, ovate to broadly so, acuminate, concolorous with inflorescence axis, bracteole narrower and thinner, both erect and appressed to ovary base; ovary 3.8–9.1 mm long, 3-angled, lorate to very narrowly elliptic in outline, pale green, sometimes purple-hued, apex weakly constricted, tepals 2.3–3.1 × 0.7–1.7 mm, shape and habit as male, inserted on the margin of a 1.8–2.5 mm diam. fleshy torus, when fresh forming an annulus in centre of flower bearing 3 0.1–0.7 mm long staminodia opposite outer tepals; style inserted in central depression, 0.9–1.2 mm long, styles 3, spreading, bifid, gynoecium concolorous with tepals. Capsule (18–)20–30 × 13–20 mm, pedicel reflexed and thus more or less erect at dehiscence, oblong-elliptic to obovate in outline, thick-chartaceous, base obtuse, apex rounded to truncate, dry and withered flowers persistent until relatively late in development (early April) on a ca 1.5–2 mm long stipe, pale brown with darker coppery-brown speckling, dehiscing apically at least at first. Seed 4.6–6.5 × 5.0–6.5 mm excluding wing, irregularly lenticular, dark brown, wing 10.5–18.8 × 6.7–8.3 mm, oblong to oblong-elliptic, winged all round margin though with elongated towards rounded to obtuse base and apex, pale brown, translucent with fine paler speckling.

##### Distribution.

South Africa, endemic to the Eastern Cape (as far west as the Winterberg) and KwaZulu Natal.

##### Vernacular name(s).

*Cunningham* 2486, a sterile specimen grown from a root bought at Umlazi Muthi market appears to be *Dioscorea rupicola* and has the name iMpinyampinya. The name inKwa may also be associated with this species.

##### Ecology.

*Dioscorea rupicola* grows in the margins of and clearings in forests and bush (including *Leucosidea sericea* woodland) and is often associated with watercourses and rocky kloofs. Archibald (1968) stated that it occurs on shady eastern and southern slopes on moist black doleritic soils in association with *Podocarpus falcatus* and *Olea capensis* in the Eastern Cape and that it is associated with and dolerite boulders. It is usually encountered from 1200 to 2100 m, but has been collected at lower altitudes in Alexandra District (*Rudatis* 1269, 600 m) and Umzinto District (*Strey* 7052, ca 580 m) in KwaZulu Natal.

##### Conservation.

*Dioscorea rupicola* is found widely in higher elevation sites in the Eastern Cape and KwaZulu Natal. Its EOO and AOO will greatly exceed the threshold for threatened IUCN categories (20, 000 km2/2000 km2) (IUCN 2001) and its provisional status is LC.

##### Uses.

None known. Like *Dioscorea multiloba*, data on the steroid content of this species is desirable.

##### Notes.

The specimens bearing labels Dioscor. 3, *Ecklon & Zeyher* 21.12 at TCD (♂ fl.) and LE (seen by Prain in 1916 according to a note on the Kew isotype) appear likely to have made from the plant material that was taken to Berlin and cultivated to yield the type. Alternatively it is possible that they were collected from other plants with that seed or tuber in late 1831 or early 1832. The locality 21.12 suggests that the collection was made in the Winterberg mountains in the Eastern Cape (Glen and Germishuisen 2010). Thus these specimens are likely to represent clonotype or paratype material.

Unpublished sequence data shows that *Dioscorea rupicola* forms a clade with the other two species covered here. Thus the shift in androecium morphology is likely to be a recent, pollinator-driven event correlated with the erect inflorescences bearing recurved flowers with erect to ascending tepals (Fig. 3C, D).

##### Specimens examined.

**South Africa. Eastern Cape:** Winterberg Mountains, ♂ fl. late 1831 or early 1832, *Ecklon & Zeyher* s.n. (TCD!, LE) (see Notes above); Collected at Mt. Kemp, Keiskamma Hoek and cultivated at Irene, ♀ fl. 20 Dec 1954, *Pole-Evans* 4847 (K!, PRE); Fort Beaufort District, Hogsback, big dolerite cliff near top of pass facing South, ♂ & ♀ fl. 10 Dec 1961, *Archibald* 7557 (K!); same locality, ♂ fl. (post anthesis) & ♀ fr. 27 Apr 1962, *Archibald* 7560 (K!); same locality, ♂ fl. 15 Nov 1961, *Archibald* 7537 (PRE!); Victoria East, on pass Port Elizabeth aspect, ♂ fl. 15 Nov 1961, *Archibald* 7551 (K!); Victoria East, about ¼ way up pass, sterile 15 Nov 1961, *Archibald* 7552 (K!); Victoria East, about ¼ way up pass, ♀ fl. 10 Dec 1961, *Archibald* 7558 (K!); Hogsback, ♂ fl. 27 Dec 1944, *Acocks* 11019 (K!); Victoria East, Hogsback Natural Forest Reserve, above Swallowtail Falls, sterile 17 Apr 1955, *Johnson* 1273 (K!, PRE); King William’s Town District, no further data, ♂ fl. 26 Jan 1956, *Comins* 1425 (K!, PRE); Mountains near Ntsizwa (in umbros. Mont. Jnsiowa), ♂ fl. 28 Jan 1895, *Schlechter* 6443 (K!, PRE!, Z digital images!); Insizwa Forest Reserve, sterile 23 Feb 1958, *Wilson & Buchner* 163 (K!); Insizwa Forest Reserve, sterile seedling 23 Feb 1958, *Wilson & Buchner* 164 (K!); Insizwa Forest Reserve, sterile seedling 23 Feb 1958, *Wilson & Buchner* 165 (K!); Insizwa Forest Reserve, sterile seedling 23 Feb 1958, *Wilson & Buchner* 166 (K!); Insizwa Forest Reserve, sterile seedling 23 Feb 1958, *Wilson & Buchner* 167 (K!); Insizwa Forest Reserve, sterile 23 Feb 1958, *Wilson & Buchner* 16 (K!); Kokstad, Tabankulu Forest, ♂ fl. Jan 1925, *Dist. Forest Officer* 558 (PRE!); Prentjiesberg, Ugie “Forest Reserve”, sterile 12 Nov 2000 *Potgieter* 392 (NU [NU0028393, 4]). (2 sterile sheets, possibly *Dioscorea rupicola* (28393) and *Dioscorea multiloba* (28394).) **KwaZulu Natal:** Alfred District, Weza, Ingeli (Ngeli) slopes, 1 Jan 1966, *Strey* 6284 (K! ♀ fl., (NU [NU0028252 digital image ♂ fl.!], PRE, UDW digital image ♂ fl. !); Alfred District, Ngeli Mountain, ♂ fl. & ♀ fl. 2 Jan 1969, *Hilliard & Burtt* 5758 (E, K!, NU [NU0028253 digital image!]); Weza State Forest, South boundary of Farm Diabolo, ♂ fl. 3 Dec 1989, *Abbott* 4583 (NH, PCE!, PRU); Griqualand East, Mount Currie, ♂ fl. Feb- Apr 1883, *Tyson* 468 (K!, Z digital image!); Griqualand East, Mount Currie, ♀ fr. Feb- Apr 1883, *Tyson* 1433 (K! Z digital image!); Kokstad, Mt. Currie slopes, ♂ fl. without date, *Dist. Forest Officer* 636/F.D. Herb 7215 (K!, PRE!); Polela District, Farm “Glengariff”, ♂ fl. late Jan 1981, *Rennie* s.n. (NU [NU0028254 digital image!]); Polela District Ndunduina Bush, Glengariff, 5 Jan 1974, *Rennie* 510 (NU [NU0028391 digital image!]); Sunset Farm, 2929DC, ♂ fl. buds 17 Jan 2000, *Rennie* 2526 (NU [NU0028259 digital image!]); Umzinto District, Ellesmere, ♂ fl. 18 Dec 1966, *Strey* 7052 (K!, NU [NU0028356 digital image!], UDW, possibly also at PCE (Ellesmere, ? Dumisa, 19 Dec 1966)); Alexandra District, Ellesmere, ♂ fl.20 Feb 1910, Rudatis 1269 (K!); Eastern Frontier C.B.S, Botha’s Hill, ♀ fl. without date, *Macowan* 537 (TCD!) Weenen, old bush above ‘Lulwers’, ♂ fl. Dec 1923 *Rogers* 28163 (Z digital image!); York-Rietvlei road, ca 1 km South of Karkloof turnoff, ♂ fl. 17 Jan 1987, *Goldblatt & Manning* 8362 (MO, NU [NU0028236 digital image!], PRE!); On Road Bulwer to Underberg-SA Paper Pulp Forest, ♀ fl. & immature fr. 22 Feb 1958, *Wilson & Buchner* 156 (K!, 4 sheets); On Road Bulwer to Drakensberg Garden, ♂ fl. 22 Feb 1958, *Wilson & Buchner* 159 (K!, 3 sheets); Drakensberg, upper Umkomaas, ♂ fl. 15 Dec 1958, *Werdermann & Oberdieck* 1395 (B, K!); Underberg District, Cobham State Forest, Emerald Vale, ♀ fl. & immature fr. 4 Mar 1985, Hilliard & Burtt 18309 (E, NU [NU282256 digital image!]); Underberg District, Cobham State Forest, Emerald Vale, ♂ fl. 14 Jan 1985, *Hilliard & Burtt* 18061 (E, NU [NU282257 digital image!], PRE); Underberg District, Sunset, Upper Lurane, ♀ fl. & immature fr. 12 Jan 1980, *Rennie* 1095 (NU [NU0028389 digital image!]); Underberg District, Sunset, Upper Lurane Valley, ♂ fl. 12 Jan 1980, *Rennie* 1094 (NU [NU0028392 digital image!]); Mpendhle District, Loteni Nature Reserve, Ngondwini valley, ♂ fl.25 Dec 1978, *Hilliard & Burtt* 11828 (K!, NU [NU0028386 digital image!]); Impendhle District, cultivated in Pretoria, sterile Feb 1962, *Roux* s.n. (K!, PRE); Bergville District, Cathedral Peak area, ♂ fl. 3 Dec 1952, *Killick* 1812 (K!, NU [NU0028399 digital image!], PRE); Lions River District, Nhluzane, ♂ fl. 28 Oct 1976, *Hilliard & Burtt* 9091 (K!, NU [NU0028388 digital image!]); Lions River District, Ross, Umgeni Poort, ♂ fl. 21 Dec 1964, *Moll* 1400 (K!, (NU [NU0028396 digital image!], PRE); Ngotshe, collected at Ngome Forest and cultivated at Irene, ♀ fl. & immature fr. 20 Dec 1954, *Pole Evans* 4852 (K!, PRE); Ngotshe District, Ngome, above forest ♀ fr. 2 Apr 1977, *Hilliard & Burtt* 9932 (K!, NU [NU0028387 digital image!]); Louwsburg District, Ngome, ♂ fl. 14 Dec 1969, *Strey* 9370 (K!, NU [NU0028397 digital image!]); Vryheid District, Nhlugatshe Mt., ♂ fl. 18 Dec 1965, *Hilliard & Burtt* 3354 (E, NU [NU0028258 digital image!]); Drakensberg, Estcourt, Cathkin Park, ♂ fl. 22 Jan 1932, *Galpin* 11744 (K!, PRE); Inanda, no further data, ♂ fl. Without date, received Apr 1881, *Wood* 1167 (K!); Champagne Castle, ♂ fl. Jan 1955 *Odhner* 44 (NU [NU0028398 digital image!]); Drakensburg, Giant’s Castle, ♂ fl. 12 Jan 1949, *Brunjis-Haylett* 20 (NU [NU0028400 digital image!]); Giant’s Castle Game Reserve, ♂ fl. 20 Dec 1987, *Cunningham* 2720 (NU [NU0028345 digital image!]).

## Supplementary Material

XML Treatment for Dioscorea buchananii

XML Treatment for Dioscorea buchananii subsp. buchananii

XML Treatment for Dioscorea buchananii subsp. undatiloba

XML Treatment for Dioscorea multiloba

XML Treatment for Dioscorea rupicola
